# Invited Reply: Modal reasoning in non-human animals: possible ways forward

**DOI:** 10.1098/rsbl.2024.0080

**Published:** 2024-06-26

**Authors:** Jan M. Engelmann, Christoph J. Völter, Mariel K. Goddu, Josep Call, Esther Herrmann, Hannes Rakoczy

**Affiliations:** ^1^ Department of Psychology, University of California, Berkeley, Berkeley, CA 94720-1650, USA; ^2^ Department of Comparative Cultural Psychology, Max Planck Institute for Evolutionary Anthropology, Leipzig 04103, Germany; ^3^ Comparative Cognition, Messerli Research Institute, University of Veterinary Medicine Vienna, Medical University of Vienna, University of Vienna, Vienna 1210, Austria; ^4^ Department of Psychology, Harvard University, Cambridge, MA 02138, USA; ^5^ School of Psychology and Neuroscience, University of St Andrews, St Andrews KY16 9AJ, UK; ^6^ Department of Psychology, University of Portsmouth, Portsmouth PO1 2UP, UK; ^7^ Department of Developmental Psychology, Georg-Elias Müller Institute of Psychology University of Göttingen, Göttingen 37073, Germany

**Keywords:** reasonings, non-human, animals, possible, forward

*Biol. Lett.* **19**: 20230179 (Published online 21st June 2023). (https://doi.org/10.1098/rsbl.2023.0179)

We thank Redshaw & Suddendorf [1] for their thoughtful comments on our recent study. We agree that there is no straightforward litmus test of the ability to represent alternative possibilities in non-verbal populations such as chimpanzees. Each individual task is more or less convincing, and most, if not all, tasks are open to potential lower-level alternative interpretations. Redshaw & Suddendorf [2] developed the ‘forked-tube task’ (based on earlier work by Robinson *et al.* and Beck *et al.* [3,4]) to investigate modal thought in non-human primates. This task poses two main cognitive challenges: (i) subjects must represent alternative possibilities (i.e. realize that the reward may emerge from the left tube or the right tube) and (ii) subjects must figure out how to act adaptively in light of these possibilities (i.e. determine that covering both tube openings guarantees success). Given these two challenges, it is possible that non-human primates’ failure in Redshaw & Suddendorf’s original ‘forked-tube task’ was not owing to problems with challenge 1 (which is dependent on modal cognition), but rather owing to problems with solving challenge 2 (which is not dependent on modal cognition; for evidence along these lines with chimpanzees and children, see [5,6]).

To test the hypothesis that minimizing task demands would improve chimpanzees’ performance, we developed an alternative experimental approach that did not require subjects to invent a novel, ecologically irrelevant action—thereby minimizing the demands associated with challenge 2 (see [5,7,8] for similar approaches in studies with human children). Specifically, we introduced a familiarization phase during which subjects learned to produce the appropriate behavioural response. Using this approach, we found evidence for modal reasoning in chimpanzees [9]. Of course, what applies to other tasks with non-verbal populations also applies to our task: leaner interpretations—such as the ones pointed out by Redshaw & Suddendorf [1]—are available. For example, we do agree that one potential confound in our experimental setup is that two tubes were present in the test condition (potentially drawing attention to both platforms), whereas only one tube was present in the control condition. This was not the case, however, in our earlier experimental setup, which also provided evidence for modal reasoning in chimpanzees [10]. This earlier study also suggests that chimpanzees’ behaviour is not explainable in terms of representing an AND relation—another suggestion made by Redshaw & Suddendorf—as chimpanzees stopped searching when they found a reward in the first box. Instead of focusing on the details of these alternative interpretations, we will use this response to sketch a possible way forward in the investigation of modal reasoning in non-human animals.

Given that there is by now a range of tasks that have been used to probe modal reasoning in non-human animals, we propose that alternative interpretations should be discussed systematically across different tasks, instead of locally for each individual task. Even considering only our own studies on modal thought, we have found evidence for this form of reasoning in chimpanzees in some tasks but not in others. Chimpanzees seem to take alternative possibilities into consideration when evaluating others’ actions [10] and when determining the location of a reward under both physical and epistemic uncertainty [9,11]. Yet, when presented with a ‘cup task’, where one reward is hidden in a location that must contain a reward and a second reward is hidden in a location that only possibly contains a reward, chimpanzees’ behaviour was not in line with modal reasoning [12].

Such patterns of partly converging and partly diverging results raise important and interesting questions: what explains this variation? Are the patterns systematic? Do tasks that converge have a common denominator related to modal reasoning that is absent in tasks that do not? Or are there additional cognitive abilities, beyond modal reasoning, that are required to perform competently in a given task?

Answering these questions requires specifying the cognitive building blocks that underlie modal reasoning [13–16]. For example, a recent theoretical proposal suggests that the capacity for modal thought is based on a combination of a basic cognitive ability—the consideration of possible extensions of representations of the actual world—with a range of other capacities, such as action planning and counterfactual thought [12]. An illustration of this approach comes from recent work in cognitive development. As with the literature on chimpanzees’ modal reasoning, there is disagreement about the presence of modal thought at different ages in human development. In experiments that use different versions of the ‘forked-tube task’, it is typically not until around 4–5 years of age that children demonstrate competent modal reasoning [2,3]. In experiments using a different experimental approach—the ‘gumball task’, where children choose between a container where they might get a desired object and a container where they will certainly obtain a desired object—even 3-year-old children reason about possibilities [7]. One account suggests that the key factor distinguishing these two tasks is not related to modal thought *per se* but rather lies in the type of agential control children have over the possible outcomes [13]. The background assumption here is that the primary form of modal thought may be thinking about agential modality—that is, thinking about what one can or might do rather than what ‘could be the case’, independent of one’s own actions [17,18]. In the ‘gumball task’ children can freely choose a course of action, and whatever possibility ‘ends up actual’ is up to them. By contrast, children in the ‘forked-tube task’ must react to possible alternative futures that are not up to them—and, as discussed above, must innovate a solution (i.e. invent a novel action) to ‘cover their bases’ to prepare for both possibilities. The fact that human children [5] and chimpanzees [9] perform better on the ‘forked-tube task’ when they have previous experiences producing the relevant action provides support for this proposal.

Returning to the chimpanzee data, we believe that the variation in performance observed across a range of experimental approaches that aim to measure modal thought presents an opportunity (i) to develop theories of the ‘building blocks’ of modal thought and how it works in different species and (ii) to design controlled experiments that carefully test between competing hypotheses. One promising way to design these experiments is adversarial collaboration, where researchers debating richer versus leaner accounts jointly design suitable tasks and agree, *a priori*, on the interpretation of different potential patterns of results. This model has been gaining prominence in developmental research (see the ManyBabies consortium) and has also recently been applied in comparative research on a question that is closely related to the one under study here (i.e. whether non-human animals engage in mental time travel, see [19]). Investigating jointly, in an *a priori* fashion (e.g. within a registered report), the patterns of performance across a battery of tasks for which alternative accounts and task analyses make testable (and potentially competing) predictions is the way forward.

## Data Availability

This article has no additional data.
